# Synergistic Proinflammatory Responses by IL-17A and Toll-Like Receptor 3 in Human Airway Epithelial Cells

**DOI:** 10.1371/journal.pone.0139491

**Published:** 2015-09-29

**Authors:** Kazutaka Mori, Tomoyuki Fujisawa, Hideki Kusagaya, Katsumasa Yamanaka, Dai Hashimoto, Noriyuki Enomoto, Naoki Inui, Yutaro Nakamura, Masato Maekawa, Takafumi Suda

**Affiliations:** 1 Second Division, Department of Internal Medicine, Hamamatsu University School of Medicine, 1-20-1 Handayama Higashi-ku, Hamamatsu 431–3192, Japan; 2 Department of Laboratory Medicine, Hamamatsu University School of Medicine, 1-20-1 Handayama Higashi-ku, Hamamatsu 431–3192, Japan; 3 Department of Clinical Pharmacology and Therapeutics, Hamamatsu University School of Medicine, 1-20-1 Handayama Higashi-ku, Hamamatsu 431–3192, Japan; University of Rochester Medical Center, UNITED STATES

## Abstract

Viral respiratory infections activate the innate immune response in the airway epithelium through Toll-like receptors (TLRs) and induce airway inflammation, which causes acute exacerbation of asthma. Although increases in IL-17A expression were observed in the airway of severe asthma patients, the interaction between IL-17A and TLR activation in airway epithelium remains poorly understood. In this study, we demonstrated that IL-17A and polyI:C, the ligand of TLR3, synergistically induced the expression of proinflammatory cytokines and chemokines (G-CSF, IL-8, CXCL1, CXCL5, IL-1F9), but not type I interferon (IFN-α1, -β) in primary culture of normal human bronchial epithelial cells. Synergistic induction after co-stimulation with IL-17A and polyI:C was observed from 2 to 24 hours after stimulation. Treatment with cycloheximide or actinomycin D had no effect, suggesting that the synergistic induction occurred without *de novo* protein synthesis or mRNA stabilization. Inhibition of the TLR3, TLR/TIR-domain-containing adaptor-inducing interferon β (TRIF), NF-κB, and IRF3 pathways decreased the polyI:C- and IL-17A/polyI:C-induced G-CSF and IL-8 mRNA expression. Comparing the levels of mRNA induction between co-treatment with IL-17A/polyI:C and treatment with polyI:C alone, blocking the of NF-κB pathway significantly attenuated the observed synergism. In western blotting analysis, activation of both NF-κB and IRF3 was observed in treatment with polyI:C and co-treatment with IL-17A/polyI:C; moreover, co-treatment with IL-17A/polyI:C augmented IκB-α phosphorylation as compared to polyI:C treatment alone. Collectively, these findings indicate that IL-17A and TLR3 activation cooperate to induce proinflammatory responses in the airway epithelium via TLR3/TRIF-mediated NF-κB/IRF3 activation, and that enhanced activation of the NF-κB pathway plays an essential role in synergistic induction after co-treatment with IL-17A and polyI:C *in vitro*.

## Introduction

Bronchial asthma is a chronic inflammatory disease of the airway involving many cells and cellular elements [1–3]. Airway inflammation in asthma is associated with hyperresponsiveness and reversible airflow obstruction, which leads to clinical manifestations such as wheezing, chest tightness, breathlessness, and coughing. Current asthma practice guidelines emphasize the importance of inhaled corticosteroids (ICSs) as an anti-inflammatory therapy, which contributes substantially to improving the quality of life and disease control in asthma patients [4, 5]. Nevertheless, even if anti-inflammatory therapy is administered, persistent airway inflammation cannot always be controlled, leading to acute exacerbation of asthma symptoms.

Viral respiratory infections have been demonstrated to be one of the most common causes of acute exacerbation in asthma patients [3, 4]. Airway epithelial cells play an important role during viral infections as a first line of defense in the lung [6]. Viral detection by intracellular receptors in the airway such as Toll-like receptors (TLR) 3, 7, or the RIG-I-like receptor (RLR), collectively called pattern recognition receptors, elicit a strong immune response [7, 8]. Polyinosinic:polycytidylic acid (polyI:C), which is a synthetic double-stranded RNA viral mimic and a ligand of TLR3, causes severe inflammation in the lung in mouse models [9]. We have recently shown that polyI:C treatment strongly induced epithelial cell-derived cytokines and anti-microbial peptides, including IL-17C, colony-stimulating factor (CSF) 3, human β-defensin (hBD) 2, and S100A12 in normal human bronchial epithelial (NHBE) cells through the TLR/TIR-domain-containing adaptor-inducing interferon-β (TRIF)/nuclear factor (NF)-κB signaling pathway [10]. PolyI:C also elicited strong inflammatory responses inducing proinflammatory cytokines, chemokines, and metalloproteases in small airway epithelial cells [11]. These findings suggest that an excessive inflammatory response in the airway epithelium during a viral infection is closely related to the exacerbation of asthma; however, the precise molecular mechanisms have not been fully elucidated.

The proinflammatory cytokine IL-17A is mainly produced by Th17 and γδT cells and is a part of the “IL-17 family” with other five members (IL-17B-F). IL-17A is well known for its protective properties during bacterial infections and its involvement in autoimmune diseases [12]. IL-17A initiates the innate host defenses and repair responses that include the induction of proinflammatory cytokines and chemokines from epithelial cells, fibroblasts, endothelial cells, chondrocytes, and adipocytes, by binding to the receptors, IL-17RA and IL-17RC [13–15]. IL-17A induces proinflammatory cytokines, chemokines (e.g., CXCL-1, -2, -3, -5, -6, CXCL8/IL-8, CCL20, and IL-19), and hBD-2 in airway epithelial cells [13–17], which enhance inflammatory responses. We have previously shown that IL-17A or Th2 cytokines also enhanced mucin (MUC) 5AC and MUC5B induction in NHBE cells [18–20], which causes the over production of mucus in chronic airway disorders such as chronic obstructive pulmonary disease (COPD) and asthma. Recently, an increased level of IL-17A in sputum and serum has been reported in patients with severe asthma [21–23], which is associated with neutrophilic airway inflammation [2, 21]. Approximately half of the mild-to-moderate asthma patients have neutrophilic airway inflammation, a disease phenotype that responds poorly to inhaled corticosteroids [24]. These findings suggest that the existence of IL-17A in the airway increases the severity of asthma and is involved in the pathophysiology of exacerbation of asthma symptoms by enhancing the inflammatory response. In viral-induced exacerbation of asthma, however, the part of IL-17A and the interplay between IL-17A and TLR3 activation have not been fully elucidated.

In this study, we examined the molecular basis of the interaction between TLR3-mediated innate immune responses and intracellular signaling of IL-17A in airway epithelial cells. We showed that IL-17A synergistically enhances polyI:C-induced expression of proinflammatory cytokines and chemokines (IL-8, CSF3, CXCL1, CXCL5, and IL-1F9) but not type I interferon (IFN-α1, -β), in NHBE cells and BEAS-2B, which is a bronchial epithelial cell line. The subsequent mechanistic study reveals a crucial role for the transcriptional factors NF-κB and interferon regulatory factor 3 (IRF3) in IL-17A/polyI:C-provoked synergistic induction in airway epithelial cells.

## Materials and Methods

### Culture conditions

Primary NHBE cells were purchased from Lonza (Catalog No. CC-2540; Basel, Switzerland). NHBE cells were seeded in 6-well plates at 1.8 × 10^4^ cells/cm^2^ in commercially available bronchial epithelial growth medium (BEGM, Lonza). The BEAS-2B (ATCC^®^ CRL-9609^™^) cell line was obtained from American Type Culture Collection (ATCC) through Summit Pharmaceuticals International Corporation (Tokyo, Japan). BEAS-2B cells were plated in 6-well plates at 0.3–1.0 × 10^4^ cells/cm^2^ in LHC-9 serum-free medium (Gibco, Grand Island, NY). Both types of cells were incubated at 37°C in a humidified atmosphere with 5% CO_2_. Submerged cells were stimulated with polyI:C and/or IL-17A. Conditioned media were collected from the cultured NHBE cells and stored at -80°C for immunoassays.

### TLR ligands and cytokine treatments

PolyI:C (high molecular weight) was purchased from Imgenex (San Diego, CA). Recombinant human IL-17A was from R&D Systems (Minneapolis, MN). Concentrations of polyI:C and IL-17A used in this study were 50 μg/ml and 10 ng/ml, respectively. In our preliminary data and previous report [10], we have performed a concentration study of polyI:C treatment (0.1–200 μg/ml) in NHBE cells. Because 50 μg/ml of polyI:C had potent stimulation in proimflammatory cytokines mRNA expression (data not shown), we chose 50 μg/ml of polyI:C treatment in the present study. Concerning the dose of IL-17A, we have previously shown that 10 ng/ml of IL-17A induced MUC5AC mRNA expression in NHBE cells, and slight decrease of stimulation was seen when dose higher than 20 ng/ml were used [18]. Thus, we selected 10 ng/ml IL-17A in this study.

### RNA isolation and real-time RT-qPCR

Total RNA was extracted using RNA the TRIzol reagent (Invitrogen, Carlsbad, CA), and stored at -80°C. Total RNA was quantified using a spectrophotometer (NanoDrop^®^ ND-1000; Thermo Scientific, Chicago, IL) before reverse transcription PCR. Preparation of first strand cDNA was performed using the ReverTra Ace^®^ qPCR RT Master Mix (TOYOBO, Osaka, Japan) from 2 μg of total RNA. The PCR mixture consisted of 10 μl of the THUNDERBIRD^®^ SYBR^®^ qPCR Mix (TOYOBO), 0.3 μM of forward and reverse primers, and the cDNA samples (total volume of 20 μl). Real-time PCR analysis was performed using the 7500 FAST Detection System (Applied Biosystems, Foster City, CA) according to the manufacturer’s instructions as described previously [10, 18, 19].Specifically, the relative amount of mRNA was calculated from comparisons between the threshold cycle (Ct) of each sample and the Ct of the housekeeping gene β-actin or glyceraldehyde-3-phosphate dehydrogenase (GAPDH). The results were presented as 2^-(Ct of gene of interest–Ct of β-actin or GAPDH)^ in arbitrary units. The list of primers used in real-time RT-qPCR analysis is described in S1 Table. A single peak on the dissociation curve was used as evidence of purity for each amplified product. There were no visible fluctuations in the Ct values of housekeeping genes from differently treated cells throughout this study (data not included).

### ELISA

To determine the concentration of the granulocyte-colony stimulating factor (G-CSF) and the IL-8 protein in the conditioned media, double-sandwich ELISAs for human G-CSF and IL-8 were performed using a Quantikine^®^ ELISA Kit (R&D Systems). Absorbance was read at 450 nm with wavelength correction at 540 nm using a microplate reader (Synergy HT, BIOTEK, Winooski, VT). All the measurements were performed in duplicate.

### Inhibitor treatments

To investigate the influence of *de novo* protein synthesis, 5 μg/ml of cycloheximide (Calbiochem by Merck KGaA, Darmstadt, Germany) was administrated together with IL-17A and/or polyI:C treatment. To explore the stability of the mRNA, the cells were stimulated with polyI:C overnight (approximately 15 hours) to induce the expression of cytokines. Then, actinomycin D (1 μg/ml; SIGMA, Saint Louis, MO) was added together with IL-17A and/or polyI:C to block further mRNA synthesis, and mRNA was harvested at different time points (0.5, 2, 6 hours) after actinomycin D treatment. BAY11-7082 (InvivoGen, San Diego, CA), an IκB-α phosphorylation inhibitor, was added 1 hour before stimulation with IL-17A, polyI:C, and co-treatment of IL-17A/polyI:C to inhibit IκB-α phosphorylation. Cycloheximide, actinomycin D, and BAY11-7082 were dissolved in dimethyl sulfoxide before use.

### Small-interfering RNA (siRNA) and transient transfection of BEAS-2B cells

The siRNA for TLR3, Toll-like receptor adaptor molecule 1 (TICAM-1, also known as TRIF), IRF3, and tumor necrosis factor receptor 1 (TNFR1) were purchased from Santa Cruz Biotechnology (Dallas, TX). NF-κB p65 siRNA and random oligomer for negative control were obtained from Ambion Biotech (Austin, TX). BEAS-2B cells were transiently transfected with siRNAs using a DharmaFECT-based transfection kit (Thermo Scientific), as described previously [10, 18, 19]. Briefly, BEAS-2B cells were transfected using transfection mix that contained 1 μM of siRNA. After 24 hours of transfection, the transfection mix was replaced with fresh LHC-9 medium. Cells were harvested 72 hours post transfection for real-time qPCR (after stimulation for 24 hours).

### Western blot analysis

Total protein lysates from different treatments were harvested using RIPA lysis buffer (ATTO Corporation, Tokyo, Japan) and quantified with a DC protein assay (Bio-Rad, Hercules, CA). Before loading, 20 μg of the cell lysate and 4× reducing sample buffer were mixed and heated at 95°C for 8 minutes. The proteins were separated on a Mini-PROTEAN^®^ TGX gel (Bio-Rad) and transferred electronically to PVDF membranes. The membranes were blocked with 3% bovine serum albumin (BSA) in 50mM Tris-buffered saline (TBS) or 5% nonfat milk for 30 minutes at room temperature before incubation with each primary antibody overnight at 4°C or 2 hours at room temperature. Then, the membranes were incubated with HRP conjugated secondary antibodies for 30 minutes at room temperature. The ECL chemiluminescence reagent was used to detect the signal bands as described previously [10] and semi-quantitative analyses using densitometry were performed using ImageJ version 1.48v (National Institutes of Health, Bethesda, MD).

#### Antibodies

Phospho-IκBα mouse monoclonal antibody (mAb) (Catalog No. #9246), phospho-IRF3 rabbit mAb (#4947), and IRF3 rabbit mAb (#4392) were purchased from Cell Signaling Technology (Boston, MA) and diluted 1:1000 in 3% BSA/TBS or 5% nonfat milk. The monoclonal anti-β-actin antibody was produced in mice (SIGMA; A5441) and used at a 1:3000 dilution.

HRP conjugated goat anti-mouse IgG (W492B) was purchased from Promega (Tokyo, Japan) and HRP-linked anti-rabbit goat IgG (#7074) was purchased from Cell Signaling Technology. The secondary antibodies were used at 1:16700 and 1:3000 dilutions, respectively.

### Statistical analysis

Measurements are described as mean ± S.E. Experiments were carried out in duplicate and repeated in at least three independent cultures. Differences among the groups were compared using one-way or two-way analysis of variance (ANOVA) and the Tukey-Kramer multiple comparison test. The *p* values that were less than or equal to 0.05 were considered statistically significant.

## Results

### Synergistic induction of a proinflammatory response following PolyI:C and IL-17A treatment in NHBE cells

**Fig 1:**
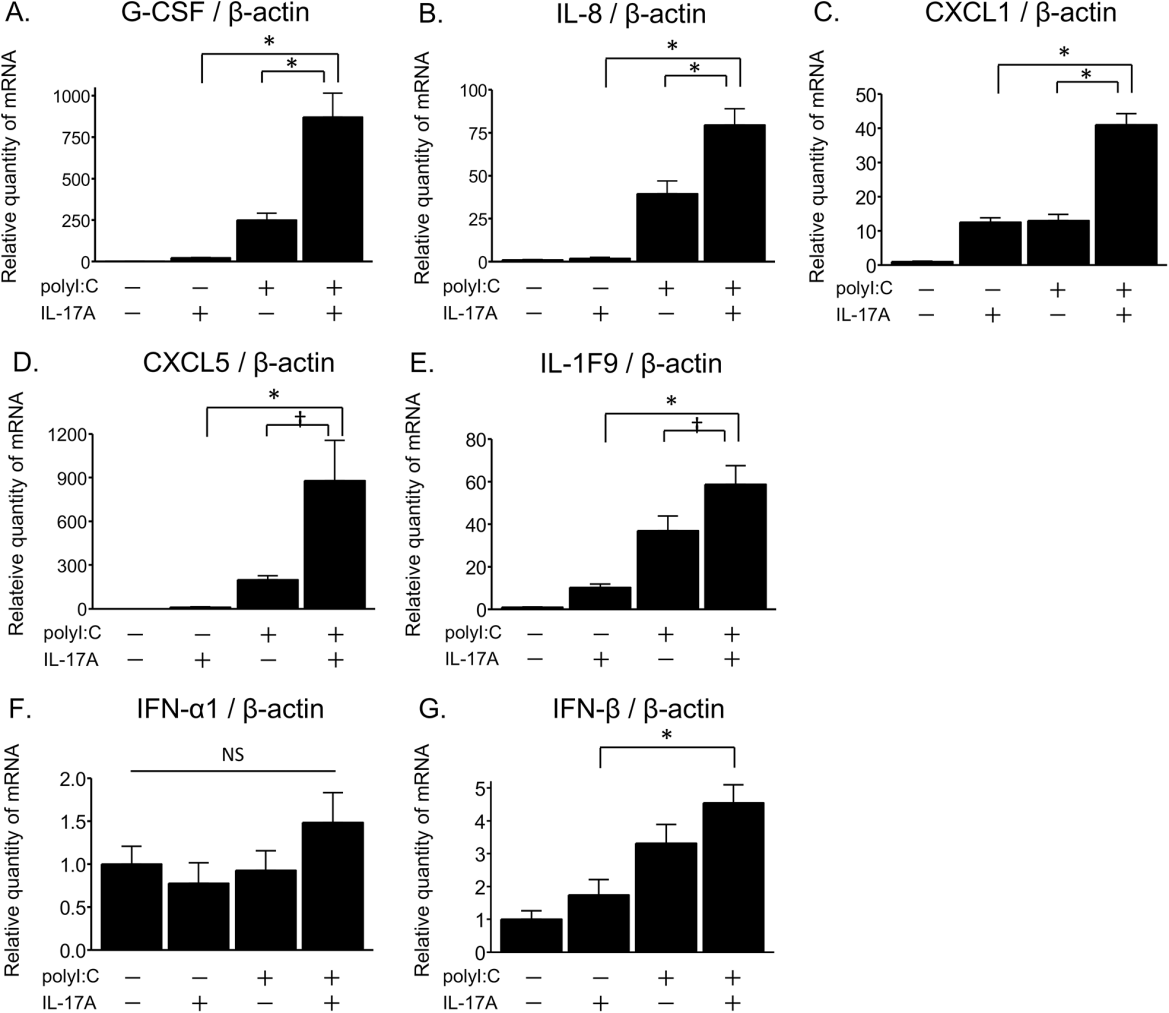
IL-17A/polyI:C-provoked synergistic induction of proinflammatory cytokines and chemokines in primary NHBE cells. At 24 hours after IL-17A (10 ng/ml) and/or polyI:C (50 μg/ml) treatment in submerged cultures, NHBE cells were harvested for RNA isolation. The mRNA levels for G-CSF (A), IL-8 (B), CXCL1 (C), CXCL5 (D), IL-1F9 (E), IFN-α1 (F), and IFN-β (G) were evaluated by real-time RT-qPCR and normalized to β-actin levels. Co-treatment with IL-17A and polyI:C synergistically up-regulated mRNA expression of G-CSF, IL-8, CXCL1, CXCL5, and IL-1F9, but not that of IFN-α1 or -β. Results are shown as the mean with S.E. from four independent cultures. * p < 0.01, † p < 0.05 versus co-treatment with IL-17A and polyI:C. NS, not significant.

To investigate the proinflammatory responses induced by viral respiratory infection in airway epithelial cells, primary NHBE cells in submerged cultures were challenged with polyI:C, which is a synthetic double-stranded RNA analogue and a ligand of TLR3, for 24 hours. The mRNA levels of cytokines and chemokines were measured by real-time RT-qPCR. The mRNA expression of proinflammatory mediators such as G-CSF, IL-8, CXCL1, CXCL5, and IL-1F9 was increased after 24 hours of exposure to polyI:C (50 μg/ml) as compared to untreated NHBE cells (Fig 1A–1E). Notably, co-stimulation with IL-17A (10 ng/ml) and polyI:C resulted in a synergistic up-regulation in G-CSF, IL-8, CXCL1, CXCL5, and IL-1F9 mRNA expression in NHBE cells (Fig 1A–1E). In terms of antiviral gene expression, polyI:C slightly induced IFN-β mRNA expression but not IFN-α1 in NHBE cells (Fig 1F and 1G). Moreover, the addition of IL-17A to polyI:C-treated cells did not cause any additional induction of type I interferon, IFN-α1 or IFN-β (Fig 1F and 1G).

**Fig 2:**
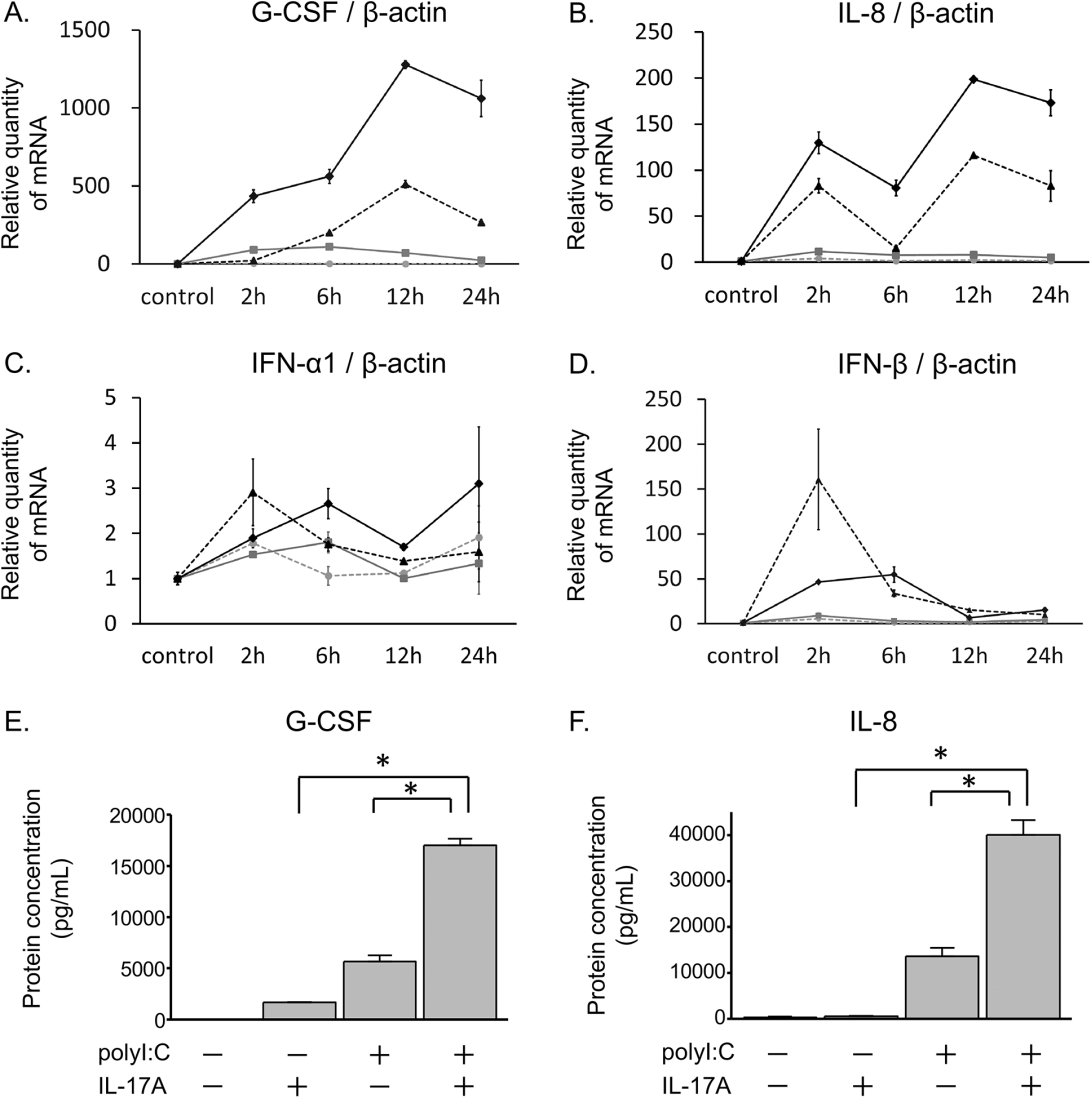
Time course analysis of mRNA expression and protein induction in NHBE cells. NHBE cells in submerged cultures were stimulated with IL-17A and/or polyI:C. Total RNA was extracted from cells at different time points (0, 2, 6, 12, and 24 hours) after treatment. G-CSF (A), IL-8 (B), IFN-α1 (C), and IFN-β (D) levels were evaluated by real-time RT-qPCR and normalized to β-actin levels. Inductions of G-CSF and IL-8 mRNA expression increased over time and was significantly higher in co-treatments with IL-17A and polyI:C than in controls or other treatments. IFN-β mRNA was significantly upregulated by polyI:C or co-treatment with IL-17A and polyI:C at 2, and 6 hours. However, IFN mRNA expression was not different between polyI:C-treatment and co-treatment with IL-17A/polyI:C. Gray dashed lines with circles, unstimulated control; gray solid lines with squares, IL-17A; black dashed lines with triangles, polyI:C; black solid lines with diamonds, co-treatment with IL-17A and polyI:C. The concentration of G-CSF (E) and IL-8 (F) proteins in the conditioned medium of submerged cultures treated with IL-17A and/or polyI:C for 24 hours were detected by ELISA. Synergistic increases in G-CSF and IL-8 in protein levels were observed after co-treatment with IL-17A and polyI:C. Results are shown as the mean with S.E. of three independent experiments. * p < 0.01 versus co-treatment with IL-17A and polyI:C.

Time course analyses indicated that the mRNA expression of G-CSF and IL-8 increased after stimulation with polyI:C (Fig 2A and 2B). Notably, co-treatment with IL-17A and polyI:C synergistically increased G-CSF and IL-8 mRNA expression from 2 to 24 hours after stimulation (Fig 2A and 2B). Although the IFN-β mRNA levels were induced at 2 hours after polyI:C stimulation, there was no synergistic increase in IFN-α1 or –β mRNA levels by co-treatment with IL-17A/polyI:C throughout the time course (Fig 2C and 2D). ELISAs demonstrated a similar trend for the synergistic induction of G-CSF or IL-8 proteins in NHBE cells after 24 hour of treatments (Fig 2E and 2F).

### IL-17A acts synergistically with polyI:C to promote G-CSF and IL-8 induction independent of *de novo* protein synthesis and mRNA stabilization

**Fig 3:**
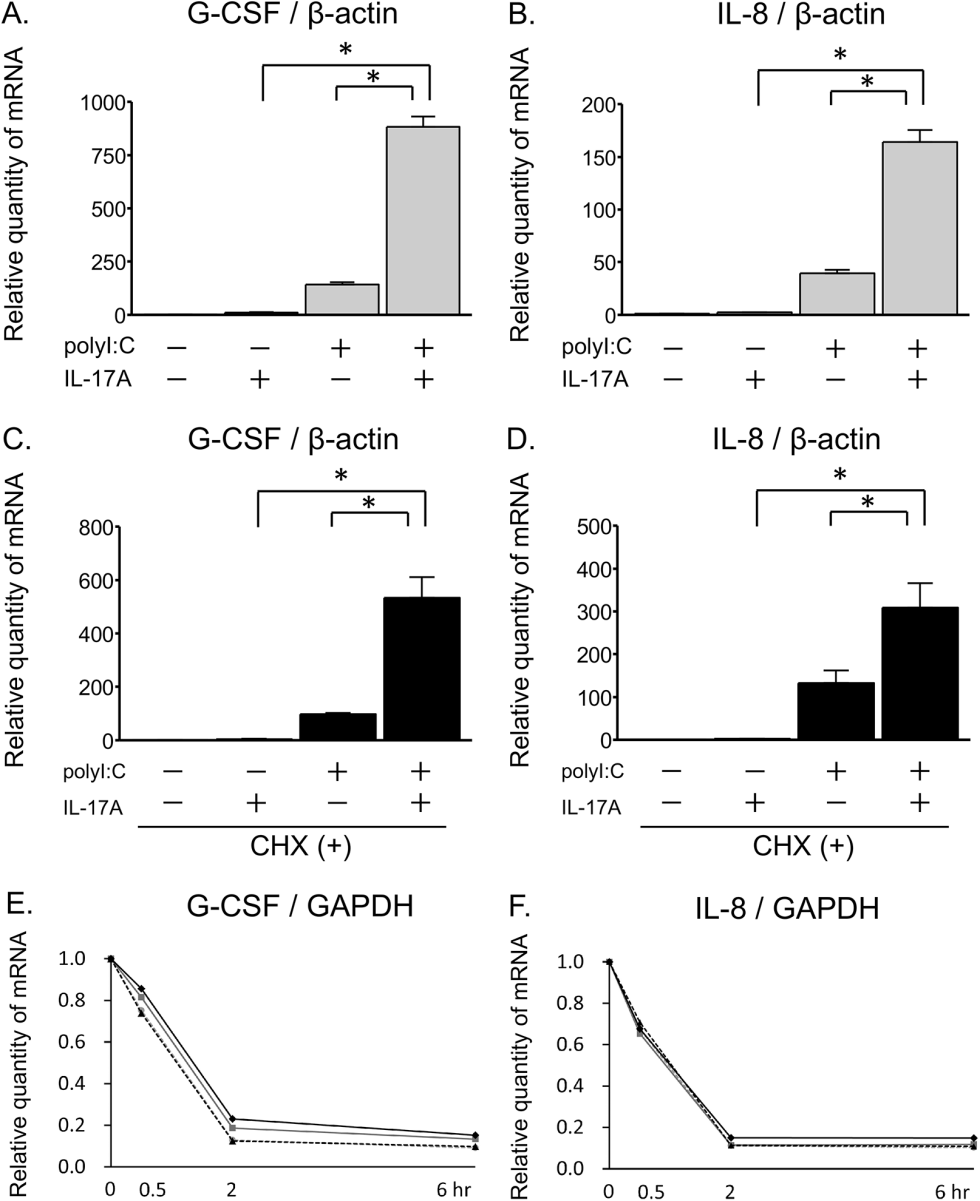
IL-17A/polyI:C-provoked synergistic induction of proinflammatory cytokines and the contribution of the posttranscriptional process. Submerged BEAS-2B cells were stimulated with IL-17A and/or polyI:C for 24 hours. G-CSF (A) and IL-8 (B) mRNA induction was evaluated by real-time RT-qPCR and normalized to β-actin levels. Co-treatment with IL-17A and polyI:C synergistically upregulated mRNA expression of these genes. Next, BEAS-2B cells were treated for 24 hours with IL-17A, polyI:C, and cycloheximide (CHX) (5 μg/ml). The mRNA levels of G-CSF (C) and IL-8 (D) were evaluated using real-time RT-qPCR and normalized to β-actin levels. CHX had no effect on the IL-17A/polyI:C-provoked synergistic induction. After overnight stimulation with polyI:C (50 μg/ml) in submerged cultures, BEAS-2B cells were incubated with actinomycin D (Act D, 1μg/ml) together with IL-17A and/or polyI:C. Total RNA was harvested at different time points (0, 0.5, 2 and 6 hours) from Act D, IL-17A and/or polyI:C treated cells. Next, G-CSF (E) and IL-8 (F) mRNA levels were evaluated by RT-qPCR and normalized to GAPDH levels. There was no significant difference between each treatment in two-way ANOVA analyses. Gray dashed lines with circles, Act D only; gray solid lines with squares, Act D + IL-17A; black dashed lines with triangles, Act D + polyI:C; and black solid lines with diamonds, Act D + IL-17A + polyI:C. Results are shown as the mean with S.E. of three independent experiments. * p < 0.01, † p < 0.05 vs. co-treatment with IL-17A and polyI:C.

Prior to performing mechanistic analyses on the synergistic induction of expression, we confirmed that the mRNA expression of G-CSF and IL-8 was also synergistically upregulated by co-treatment with IL-17A and polyI:C in BEAS-2B cells, which are an epithelial cell line from the human bronchus, as well as NHBE cells (Fig 3A and 3B). To assess whether some *de novo* protein synthesis was involved in the IL-17A/polyI:C-provoked synergistic induction of proinflammatory cytokines, BEAS-2B cells were treated with the protein synthesis inhibitor cycloheximide in the presence of IL-17A and polyI:C. Cycloheximide had no effect on the IL-17A/polyI:C-provoked synergistic induction of G-CSF and IL-8 mRNA levels (Fig 3C and 3D). To determine whether mRNA stability was related to the synergy, BEAS-2B cells were stimulated with polyI:C overnight and treated with IL-17A and/or polyI:C in the presence of actinomycin D. The relative amount of G-CSF and IL-8 mRNA significantly decreased over time after the addition of actinomycin D, and IL-17A and/or polyI:C treatment had no effect on the persistence of these mRNAs (Fig 3E and 3F). Taken together, these results indicate that the IL-17A/polyI:C-induced synergistic proinflammatory gene expression occurred without any participation of *de novo* protein synthesis or mRNA stabilization.

### IL-17A/polyI:C-provoked synergistic induction was mediated by the TRIF, NF-κB, and IRF3 pathways

We next evaluated the signaling pathways and transcription factors contributing to the IL-17A/polyI:C-provoked synergistic induction of G-CSF and IL-8. We first ascertained the involvement of the TLR3-TRIF (also known as TICAM-1) axis using TLR3 and TICAM-1 siRNAs. TRIF/TICAM-1 is an adapter in responding to activation of TLR3. BEAS-2B cells were transiently transfected with siRNAs for TLR3 or TICAM-1.

**Fig 4:**
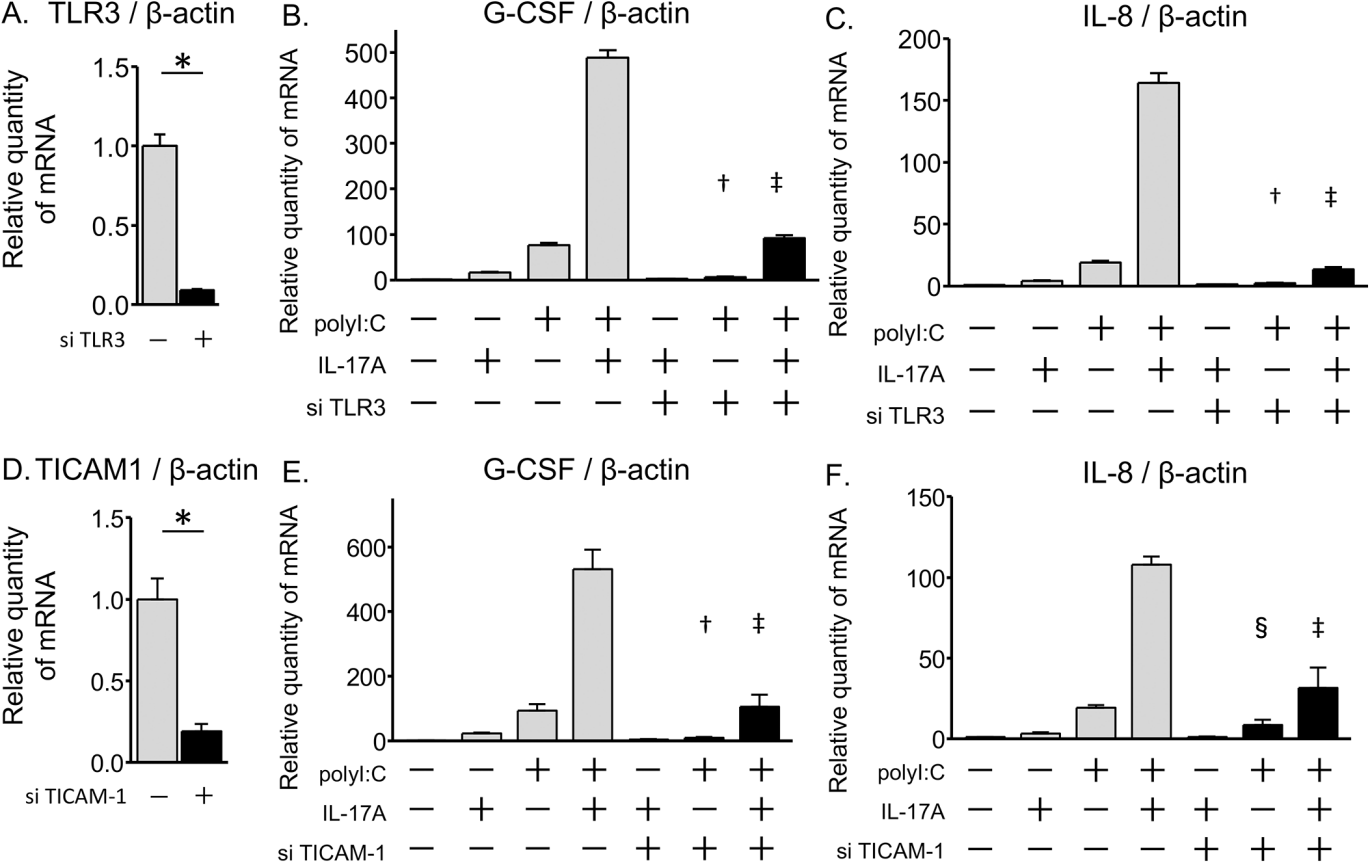
Contribution of the TLR3—TRIF axis in the IL-17A/polyI:C-provoked synergistic induction of gene expression. Transient transfection of BEAS-2B cells was performed using a TLR3 siRNA, a TICAM-1 siRNA or a random oligomer. At 24 hours after transfection, the transfection mix containing siRNAs was replaced with fresh LHC-9 medium. After 2 days from transfection, the cells were treated for 24 hours with IL-17A and/or polyI:C. mRNA levels of G-CSF, IL-8, TLR3, and TICAM-1were evaluated using quantitative real-time PCR. TLR3 siRNA (solid bars) attenuated its own mRNA level as compared with the random oligomer negative control (shadowed bars) (A). PolyI:C-induced G-CSF and IL-8 mRNA expression was suppressed by TLR3 siRNAs, and IL-17A/polyI:C-provoked synergistic induction was attenuated (B, C). TICAM-1 siRNA (solid bars) suppressed 80% of its own message compared with the negative control (shadowed bars) (D). PolyI:C-induced mRNA expression of G-CSF and IL-8 was suppressed by TICAM-1 siRNAs, and synergistic mRNA induction of G-CSF and IL-8 by co-treatment with IL-17A and polyI:C was significantly attenuated (E, F). All measurements were averaged from duplicate samples, and the experiment was repeated three times. * p < 0.001, siRNA vs. negative control. † p < 0.01, siRNA vs. negative control in polyI:C treatment. ‡ p < 0.01, siRNA vs. negative control in co-treatment with IL-17A and polyI:C. § p < 0.05, siRNA vs. negative control in polyI:C treatment.

The TLR3 siRNA suppressed 90% of its own message (Fig 4A). At 72 hours post transfection, mRNA was harvested after 24 hours of stimulation with IL-17A and/or polyI:C. TLR3 siRNA significantly attenuated polyI:C-induced G-CSF and IL-8 expression, and synergistic induction by co-treatment with IL-17A/polyI:C (Fig 4C and 4D).The TICAM-1 siRNA was effective in attenuating its own message (Fig 4D). At 2 days post transfection, cells were stimulated for 24 hours with IL-17A and/or polyI:C. The TICAM-1 siRNA clearly attenuated the polyI:C-induced G-CSF and IL-8 expression, and IL-17A/polyI:C-provoked synergistic induction of these genes (Fig 4E and 4F). These results demonstrated that TLR3-TRIF was involved in both the polyI:C-induced gene expression and the IL-17A/polyI:C-induced synergistic gene expression in airway epithelial cells.

**Fig 5:**
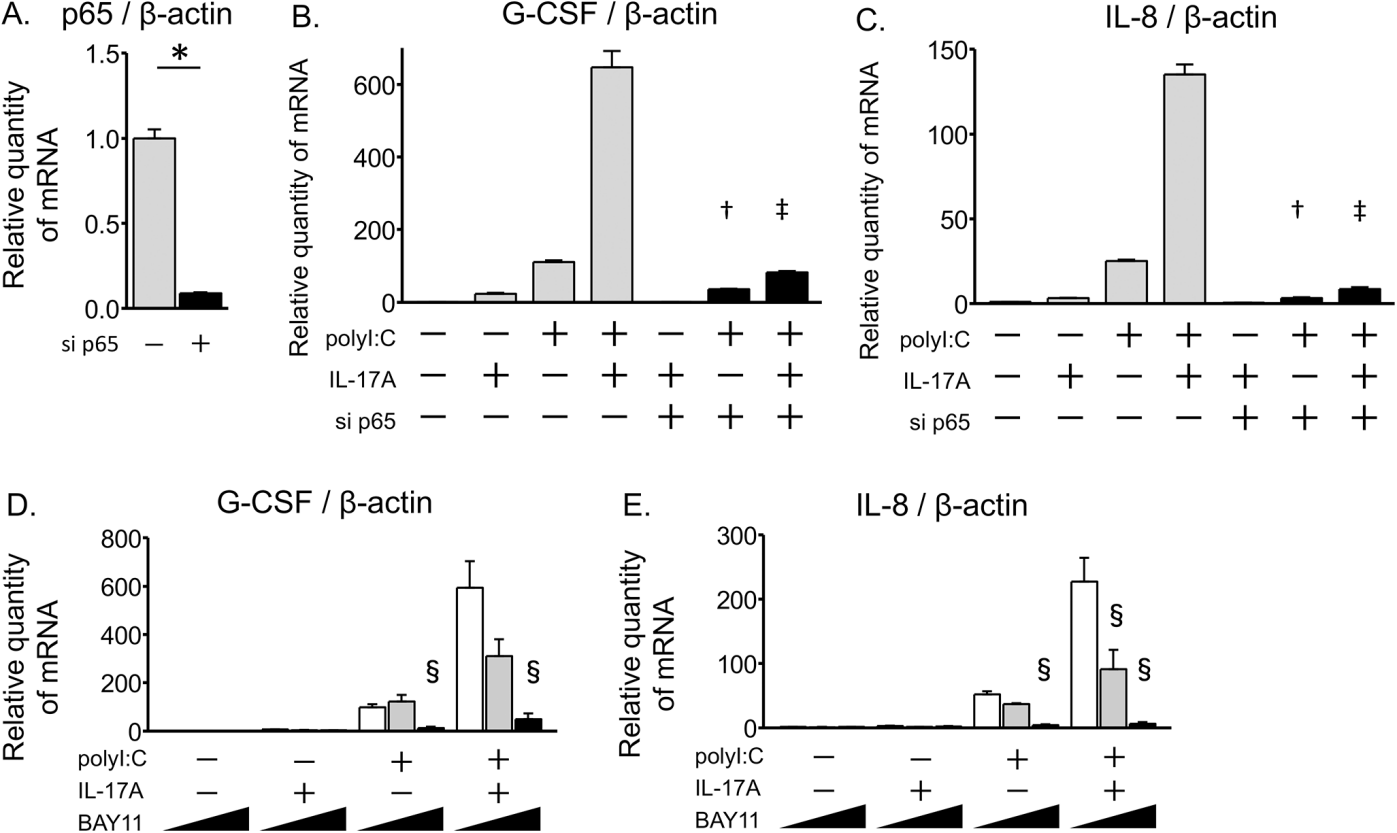
IL-17A/polyI:C-provoked synergistic induction of proinflammatory cytokines depends on the NF-κB pathway. BEAS-2B cells were transfected with siRNA for NF-κB p65 (solid bars) or a negative control (shadowed bars) for 24 hours. At 2 days after exposure to siRNA, transfected cells were treated for 24 hours with IL-17A and/or polyI:C, and then the expression of mRNA was evaluated by quantitative real-time PCR. Treatment with p65 siRNA reduced endogenous p65 mRNA levels about 90% as compared with the random oligomer negative control (A). PolyI:C-induced G-CSF and IL-8 mRNA expression was suppressed by p65 siRNA, and the IL-17A/polyI:C-provoked synergistic induction was strongly attenuated (B, C). BEAS-2B cells were pre-incubated in the presence of the IκBα inhibitor BAY11-7082 (0–10 μM) for 1 hour and treated with IL-17A and/or polyI:C in submerged cultures. After 24-hour treatments, total RNA was harvested, and G-CSF (D) and IL-8 (E) mRNA expression was evaluated using quantitative real-time PCR that was normalized to β-actin levels. BAY11-7082 treatment inhibited IL-17A/polyI:C-provoked synergistic G-CSF and IL-8 induction in a concentration-dependent manner. All measurements were averaged from duplicate wells, and the experiment was repeated three times. * p < 0.001, p65 siRNA vs. negative control. † p < 0.01, p65 siRNA vs. negative control in polyI:C treatment. ‡ p < 0.01, p65 siRNA vs. negative control in co-treatment with IL-17A and polyI:C. § p < 0.05, vs. BAY11-7082 absent.

Next, we examined the role of the NF-κB pathway, which is related to signaling pathways downstream of both TLR3 and IL-17A, using a p65 siRNA. The p65 siRNA attenuated 90% of its own message (Fig 5A). The IL-17A/polyI:C-induced synergistic increases in expression of G-CSF and IL-8 mRNA levels were strongly suppressed by the p65 siRNA as compared to the negative control (Fig 5B and 5C). Comparing the induction levels between IL-17A/polyI:C co-treatment and polyI:C treatment alone, p65 siRNA significantly decreased the amplification ratio (co-treatment/polyI:C) of both G-CSF and IL-8 mRNA expression as compared to the negative control (mean, 5.87 to 2.29, 5.37 to 2.62, respectively; S2 Table). To confirm the impact of the inhibition of the NF-κB pathway, submerged BEAS-2B cells were pre-incubated with BAY11-7082 (5–10 μM), an IκB-α phosphorylation inhibitor, for 1 hour before stimulation and then the cells were stimulated for 24 hours with IL-17A and/or polyI:C in the presence of BAY11-7082. BAY11-7082 treatment inhibited the IL-17A/polyI:C-provoked synergistic G-CSF and IL-8 induction in a dose-dependent manner (Fig 5D and 5E). These results indicate that the NF-κB pathway was indispensable for the IL-17A/polyI:C-provoked synergistic G-CSF and IL-8 mRNA induction in airway epithelial cells.

**Fig 6:**
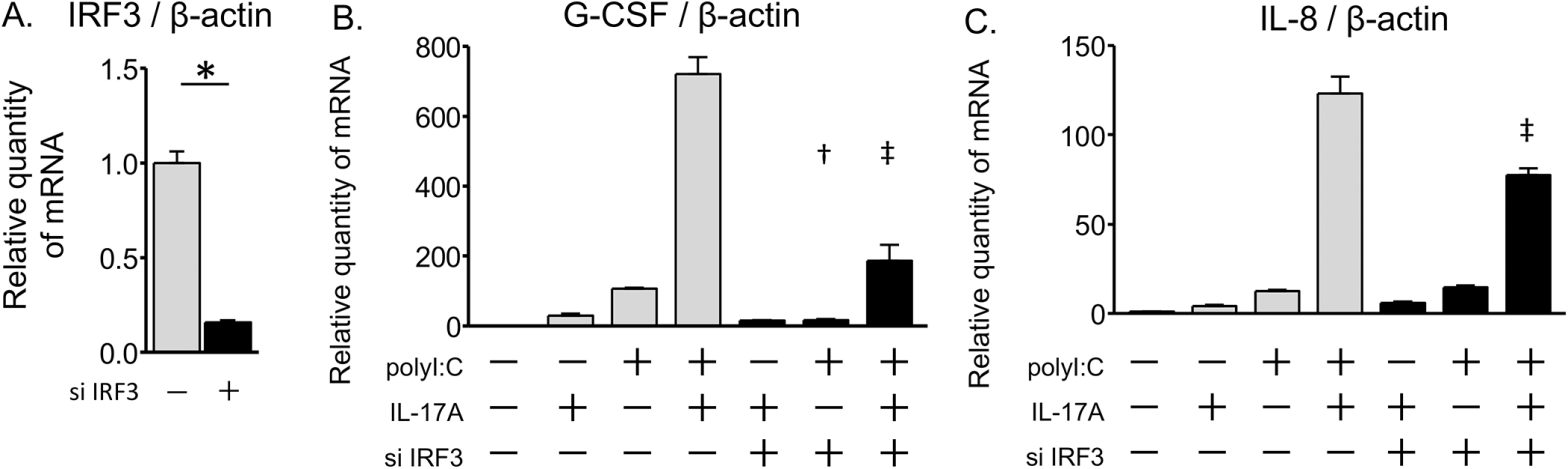
Effects of IRF3 siRNA on inflammatory cytokines gene expression. BEAS-2B cells were transfected with IRF3 siRNA (solid bars) or a random oligomer negative control (shadowed bars). After 24 hours of transfection, the siRNA transfection mix was replaced with a fresh LHC-9 medium. At 2 days after siRNA treatment, transfected cells were incubated for 24 hours with IL-17A and/or polyI:C and mRNA expression was evaluated by real-time RT-qPCR. The IRF3 siRNA reduced endogenous IRF3 mRNA expression (A). Synergistic mRNA induction of G-CSF (B) and IL-8 (C) was significantly suppressed by the siRNA knockdown of IRF3. All measurements were averaged from duplicate wells, and the experiment was repeated three times. * p < 0.001, IRF3 siRNA vs. negative control. † p < 0.01, IRF3 siRNA vs. negative control in polyI:C treatment. ‡ p < 0.01, IRF3 siRNA vs. negative control in co-treatment with IL-17A and polyI:C.

To further confirm the involvement of IRF3 in the IL-17A/polyI:C-induced synergistic proinflammatory response, we used an siRNA approach to knock down IRF3. As shown in Fig 6A, IRF3 siRNA was effective in reducing IRF3 expression 80% of its original level. The IRF3 siRNA treatment also suppressed the IL-17A/polyI:C-provoked induction of G-CSF and IL-8 (Fig 6B and 6C). The IRF3 siRNA decreased the amplification ratio (co-treatment/polyI:C) of IL-8 mRNA expression (mean, 9.94 to 5.26, S2 Table), however, there was no reduction observed in the amplification ratio of G-CSF mRNA expression (mean, 6.78 to 11.0, S2 Table), which suggests that the IRF3 pathway was involved in the synergy of IL-8 mRNA expression but was not in the synergy of G-CSF mRNA expression. The detailed mechanism of IL-17A/polyI:C-provoked synergistic G-CSF expression and that of IL-17A/polyI:C-provoked synergistic IL-8 expression may be slightly different. These results indicate that the IRF3 pathway is partially involved in the IL-17A/polyI:C-induced synergistic proinflammatory response in airway epithelial cells, on the other hand, the NF-κB pathway is more important for the synergism than the IRF3 pathway.

### TNF receptor signaling did not contribute to IL-17A/polyI:C-provoked synergistic induction

**Fig 7:**
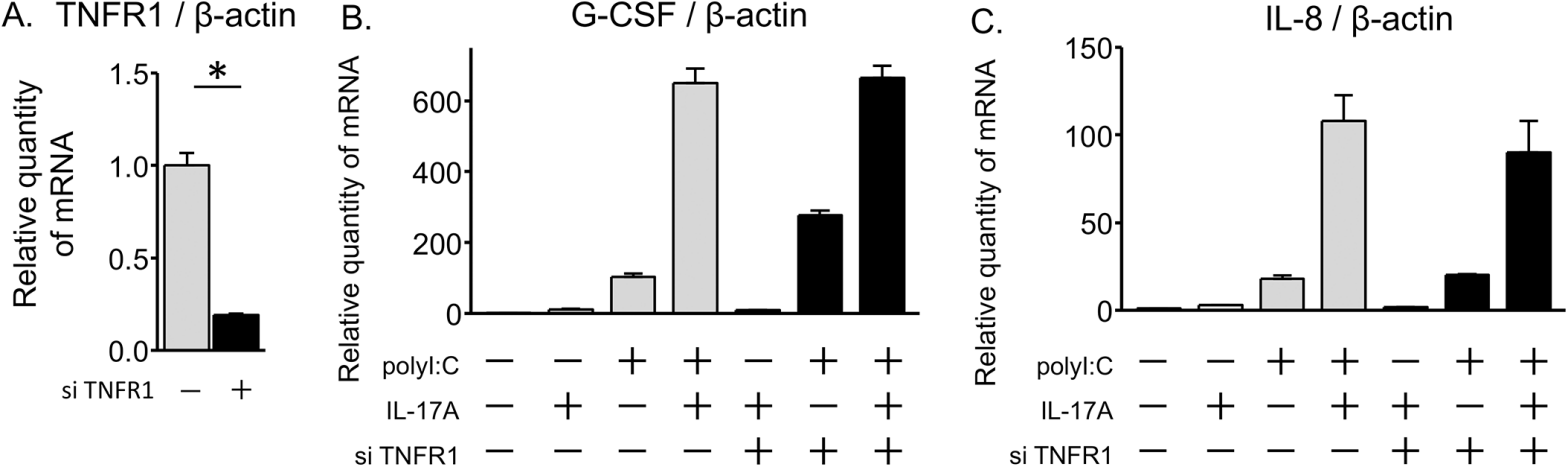
Blocking of TNF receptor signaling did not affect IL-17A/polyI:C-induced proinflammatory cytokine gene expression. BEAS-2B cells were transfected with TNFR1 siRNA (solid bars) or a random oligomer negative control (shadowed bars) for 24 hours. At 72 hours after transfection, transfected cells were harvested for real-time quantitative PCR analysis following 24 hours of stimulation with IL-17A and/or polyI:C. Treatment with TNFR1 siRNAs reduced endogenous TNFR1 mRNA level about 80% as compared with the negative control (A). Expression of G-CSF and IL-8 mRNAs that were induced by polyI:C or co-treatment with IL-17A/polyI:C was not attenuated by knockdown of TNFR1 (B, C). * p < 0.001, TNFR1 siRNA vs. negative control.

To date, several groups have demonstrated the synergistic induction of proinflammatory cytokines by IL-17A and tumor necrosis factor (TNF) α treatment [25, 26]. We investigated whether TNF receptor signaling is involved in IL-17A/polyI:C-induced synergistic proinflammatory responses using siRNAs for TNF receptor 1 (TNFR1). Although the TNFR1 siRNA was effective in attenuating its own mRNA expression 80% of its original level (Fig 7A), transfection of TNFR1 siRNA did not have any effect on G-CSF and IL-8 mRNA expression that was induced by IL-17A and/or polyI:C treatment (Fig 7B and 7C). These results indicate that TNF receptor signaling did not contribute to the IL-17A/polyI:C-induced synergistic proinflammatory response in airway epithelial cells.

### Activation of NF-κB and IRF3 by treatment with polyI:C and/or IL-17A

**Fig 8:**
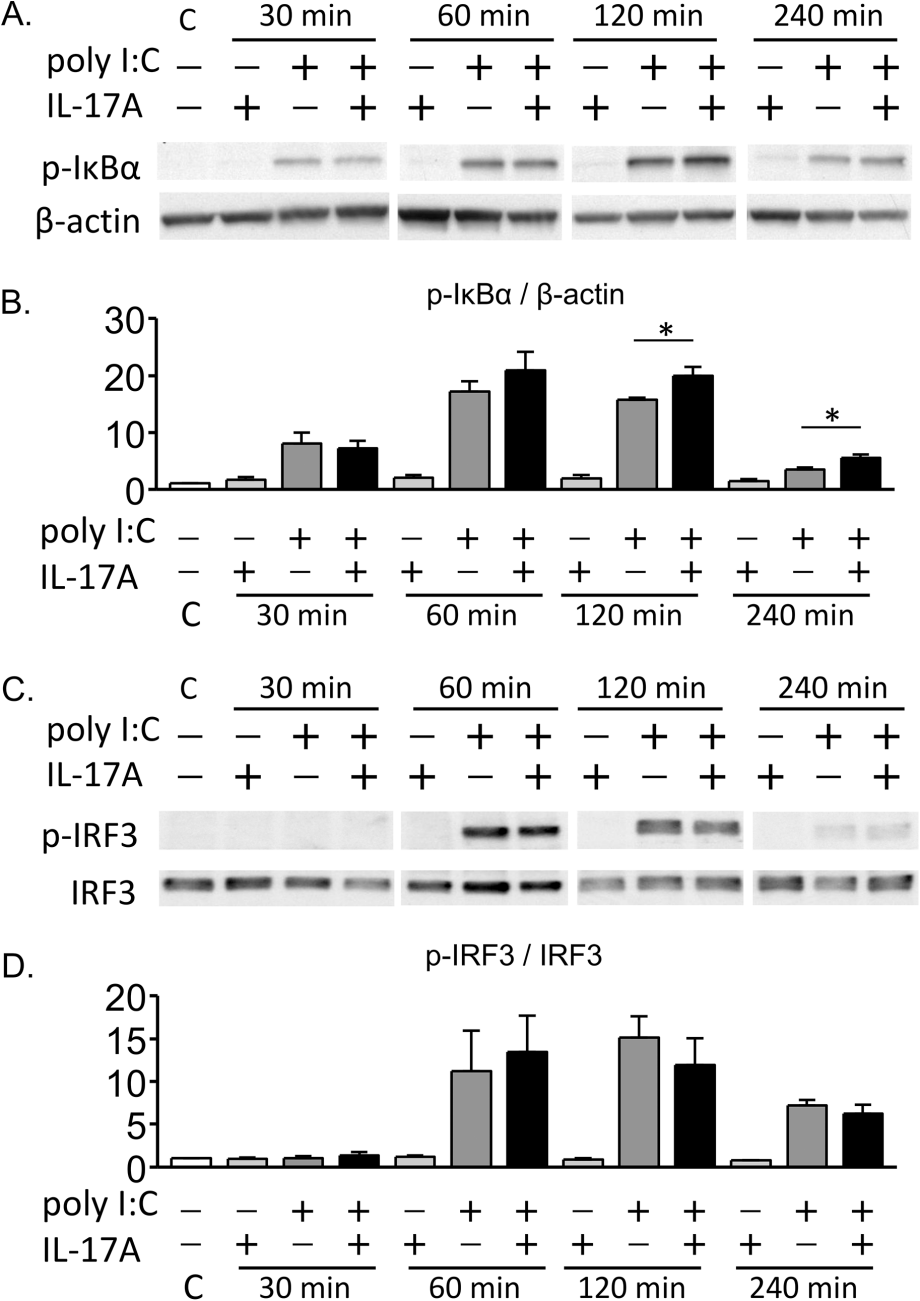
Activation of NF-κB and IRF3 signaling pathways after treatment with IL-17A and/or polyI:C. BEAS-2B cells were stimulated with IL-17A and/or polyI:C in submerged cultures. Whole cell lysates were obtained at different time points (30, 60, 120 and 240 minutes) after treatment. Phosphorylation of IκBα was assessed by western blotting. β-actin was used as a loading control. Band images from the representative experiment (A) and semi-quantification of phosphorylated IκBα using densitometry (B) are shown. IκB-α phosphorylation was strongly induced at 30–240 minutes in cells treated with polyI:C alone or co-treated with IL-17A and polyI:C. The relative amount of IκB-αphosphorylation was significantly higher in co-treatment conditions with IL-17A and polyI:C as compared to polyI:C treatment alone at 120 and 240 minutes (p = 0.036 and 0.044, respectively). Phosphorylation of IRF3 was also assessed by western blotting. The representative experiment (C) and densitometric analysis (D) are shown. IRF3 phosphorylation was enhanced by polyI:C treatment or co-treatment with IL-17A and polyI:C, and no difference in the relative amount of IRF3 phosphorylation was observed between polyI:C treatment alone and co-treatment with IL-17A and polyI:C. Results of densitometoric analysis represent the mean with S.E. from three independent experiments. C, control. * p < 0.05, polyI:C vs. co-treatment with IL17-A and polyI:C.

To evaluate the activation of NF-κB signaling, we performed western blotting analysis to detect phosphorylation of IκB-α. BEAS-2B cells were cultured in submerged conditions until the cells were 90% confluent, and then stimulated with IL-17A and/or polyI:C. PolyI:C treatment or co-treatment with IL-17A and polyI:C induced IκB-α phosphorylation from 30 to 240 minutes after treatment (Fig 8A, S1 and S2 Files). In semi-quantitative analyses using densitometry, the relative amount of IκB-α phosphorylation was significantly higher in co-treatment conditions with IL-17A and polyI:C as compared to the single treatments at 120 and 240 minutes (Fig 8B).

Phosphorylation of IRF3 was also analyzed to assess the activation of IRF3. PolyI:C treatment or co-treatment conditions with IL-17A and polyI:C induced IRF3 phosphorylation at 60 to 120 minutes after treatment (Fig 8C, S3 and S4 Files). No significant difference was found in the relative amount of IRF3 phosphorylation between the single treatment and co-treatment conditions with IL-17A and polyI:C (Fig 8D). These results suggest that activation of both the NF-κB and the IRF3 signaling pathways were essential in polyI:C/IL-17A-induced proinflammatory responses. Moreover, the NF-κB pathway may play a primary role in mediating the synergistic induction of inflammatory genes after co-treatment with IL-17A and polyI:C in airway epithelial cells.

## Discussion

In this study, we investigated the interaction between IL-17A and TLR3 signaling in airway epithelial cells, and the regulatory mechanism of the proinflammatory response mediated by IL-17A and polyI:C, a ligand of TLR3. We found that IL-17A and polyI:C synergistically induced proinflammatory cytokines and chemokines expression (G-CSF, IL-8, CXCL1, CXCL5, and IL-1F9), but not antiviral gene expression (IFN-α1 and –β) in primary cultures of NHBE cells. The IL-17A/polyI:C-induced synergistic proinflammatory response occurred in the absence of *de novo* protein synthesis and mRNA stabilization. Attenuation of TLR3, TICAM-1 (also known as TRIF), NF-κB, and IRF3 using specific inhibitors or siRNAs decreased the synergistic effects on G-CSF and IL-8 mRNA expression. Comparing the ratio of mRNA induction between co-treatment with IL-17A/polyI:C and treatment with polyI:C alone, blocking the NF-κB pathway significantly attenuated the synergism. Western blotting analysis revealed that both NF-κB and IRF3 activation were observed in polyI:C single treatment and co-treatment with IL-17A and polyI:C. In addition, NF-κB activation was potentiated by co-stimulation with IL-17A and polyI:C as compared with the single treatment. These findings provide evidence that IL-17A and TLR3 signaling cooperate to enhance the expression of proinflammatory cytokines in the airway epithelium via TLR3/TRIF-mediated NF-κB/IRF3 activation; moreover, enhanced NF-κB dependent pathway activation may play a key role in the synergism.

Our data demonstrates that IL-17A and TLR3-signaling synergistically enhanced proinflammatory cytokine and chemokine expression in airway epithelial cells. Viral respiratory infections activate innate immune responses through pattern recognition receptors, particularly TLRs and RLRs, in airway epithelial cells [3, 7, 27], which causes exacerbation of chronic airway disorders (e.g., asthma and COPD). Patients with severe asthma are highly susceptible to viral infections leading to acute exacerbation of asthma symptoms [4], and have a higher level of IL-17A in induced sputum and bronchial biopsies [23]. The airway epithelial lining is the first line of defense; thus, the response of airway epithelial cells during viral infections is likely to be related to the pathogenesis of acute exacerbation of asthma. When allergen challenge was followed by polyI:C exposure in an asthma mouse model, there were similarities to the observations made in viral-induced exacerbation of human asthma. Mahmutovic-Persson and colleagues [28] demonstrated that airway polyI:C challenge in an allergic experimental asthma mouse model produced an exacerbation-like condition, with increased lung tissue inflammation and increased levels of neutrophils and CXCL1 expression in bronchoalveolar lavage (BAL). In this view, the present observation of the synergistic expression of G-CSF, IL-8, CXCL1, and CXCL5 after co-treatment with IL-17A and polyI:C in NHBE cells indicates a significant role of airway epithelial cells in promoting excessive neutrophilic inflammation in viral-induced exacerbations of asthma. In addition, a recent study using human skin fibroblasts showed that a combined IL-17A/polyI:C treatment resulted in the synergistic upregulation of IL-6 and IL-8 expression [29]. These results, including our data, highlight the impact of synergism for IL-17A and polyI:C in promoting excessive inflammation during viral infections. In asthma, this synergism is likely to be associated with viral infection-induced acute exacerbation, and may therefore be a therapeutic target for preventing the exacerbation of asthma.

To date, few studies describe the interaction between IL-17A and TLR signaling in airway epithelial cells, and the molecular mechanism governing the synergism elicited by IL-17A and TLRs has not been fully elucidated. Wiehler *et al*. [30] has shown the synergism of IL-17A and rhinovirus infection in NHBE cells, demonstrating that IL-17A enhanced human rhinovirus-induced IL-8 and hBD2 expression. However, their study did not mention the involvement of TLR3 and the downstream signaling molecules (e.g., TRIF, NF-κB, and IRF3). In the present study, we provide evidence that IL-17A interacts with the TLR3/TRIF signaling pathway to augment proinflammatory gene expression in a primary cultures of NHBE cells, and that the activation of downstream transcriptional factors, NF-κB and IRF3, was required in the synergistic proinflammatory response. Concerning other TLRs, Mizunoe and colleagues [31] reported that IL-17A enhanced the production of IL-8 induced by peptidoglycan (TLR2 agonist) or lipopolysaccharide (TLR4 agonist) in bronchial epithelial cells from cystic fibrosis patients, but not in NHBE cells. The interplay between IL-17A and TLR signaling may differ among disease conditions and cell types. Further studies are warranted to elucidate the molecular details of this process.

Synergism between IL-17A and other cytokines, including TNF-α, IL-1β, IL-4, and IL-13, in inflammatory gene expression has been appreciated in various cell types [26, 32–36]. A variety of mechanisms, including the action of transcription factors, was reported to be involved in the synergism [34, 36]. Our siRNA knockdown results highlight the importance of NF-κB and IRF3 activation in polyI:C-induced and IL-17A/polyI:C-induced synergistic G-CSF and IL-8 expression in airway epithelial cells. NF-κB and IRF3 are key transcription factors that are downstream of the TLR3/TRIF signaling pathway; thus, it is understandable that both NF-κB and IRF3 are involved in the induction. Moreover, comparing the induction levels between treatment with polyI:C alone and co-treatment with IL-17A/polyI:C, the blockade of the NF-κB pathway significantly attenuated the IL-17A/polyI:C-provoked synergistic induction of G-CSF and IL-8 mRNA. In addition, western blotting analysis revealed that combined treatment with IL-17A and polyI:C significantly augmented NF-κB activation, although the difference of IκB-a phosphorylation between polyI:C treatment and co-treatment with polyI:C/IL-17A was modest. These findings suggest a critical role for the NF-κB dependent pathway in the IL-17A/polyI:C-induced synergistic proinflammatory cytokine expression. Considering the broad nature of NF-κB activation, changes in NF-κB binding ability, or an interplay between NF-κB and IRF3 may be involved in the mechanisms of IL-17A/TLR3-mediated synergism, which will be a topic for future studies. In terms of other transcription factors, Huang and colleagues [34] has shown that IL-17A and IL-4/IL-13 synergistically upregulated IL-19 expression in airway epithelial cells through STAT6-dependent mechanisms.

Several groups have shown the synergistic proinflammatory cytokines expression induced by IL-17A and TNF-α treatment [25, 26]. The ability of IL-17A in combination with TNF-α to promote enhanced mRNA stability for cytokines and chemokines, including IL-8, has been previously shown in fibroblasts and airway smooth muscle cells [33, 37]. In our system, IL-17A had no effect on mRNA stability in IL-17A/polyI:C-induced synergistic G-CSF and IL-8 expression. In addition, treatment with cycloheximide had no effect on the synergy, suggesting that *de novo* protein synthesis is dispensable for the synergism. Moreover, blocking TNF receptor signaling did not attenuate the IL-17A/polyI:C-induced synergistic expression. Based on these findings, IL-17A/polyI:C-induced synergistic G-CSF and IL-8 expression in airway epithelial cells is mediated by transcriptional activation, possibly through NF-κB and IRF3 activation. The different mechanism by which IL-17A augments proinflammatory gene expression may be partly explained by the different cell types and stimulants used in each study.

The detailed molecular mechanisms of synergistic inflammatory cytokine induction may be considerably complicated. Recently, Qiao *et al*. [38] demonstrated that IFN-γ promotes chromatin remodeling to increase chromatin accessibility and augment TLR4-induced inflammatory gene expression in primary human macrophages. IFN-γ induced sustained occupancy of STAT1 and IRF1, and associated histone acetylation at the promoter and enhancer, which increased recruitment of TLR4-induced transcriptional factors, such as NF-κB and C/EBPβ, and transcription of inflammatory cytokine genes. In this study, we did not evaluate the participation of chromatin remodeling and histone acetylation in IL-17A/polyI:C-induced synergistic inflammatory cytokine gene expression, however, it is feasible that similar mechanisms exist for the synergism between IL-17A and TLR3 signaling in airway epithelium, which may augment NF-κB activation and transcription of these genes.

In summary, our data provide an explanation for the IL-17A/polyI:C-induced synergistic proinflammatory cytokine and chemokine expression in airway epithelial cells. IL-17A and polyI:C synergistically induced proinflammatory (G-CSF, IL-8, CXCL1, CXCL5, and IL-1F9) but not antiviral (IFN-α1 and -β) gene expression in primary cultured NHBE cells, which promotes the attraction of other immune cell types (e.g., neutrophils) in the airway and excessive airway inflammation. Analysis of regulatory mechanisms for IL-17A/polyI:C-induced synergistic proinflammatory responses revealed the importance of TLR3/TRIF-mediated NF-κB/IRF3 activation; moreover, enhanced activation of the NF-κB dependent pathway may play an essential role in the observed synergism. Patients with severe asthma are highly susceptible to viral respiratory infection, which results in severe airway inflammation and acute exacerbation of asthma. This study highlights an important interaction between IL-17A and TLR3 signaling in excessive airway inflammation, and control of IL-17A/TLR3-mediated NF-κB/IRF3 activation may be a novel therapeutic target to prevent exacerbation of asthma.

## Supporting Information

S1 File The original western blot image of phosphorylated IκBα. (TIF)

S2 File The original western blot image of β-actin. (TIF)

S3 File The original western blot image of phosphorylated IRF3. (TIF)

S4 File The original western blot image of total IRF3. (TIF)

S1 Table The list of primers used in real-time RT-qPCR analysis. (DOCX)

S2 Table Induction ratio between co-treatment with IL-17A / polyI:C and polyI:C. (DOCX)
