# Supplementary figures and images for: Synergistic Proinflammatory Responses by IL-17A and Toll-Like Receptor 3 in Human Airway Epithelial Cells
Source: PLoS One. 2015 Sep 29;10(9):e0139491. doi: 10.1371/journal.pone.0139491 (PMC4587973; doi:10.1371/journal.pone.0139491)

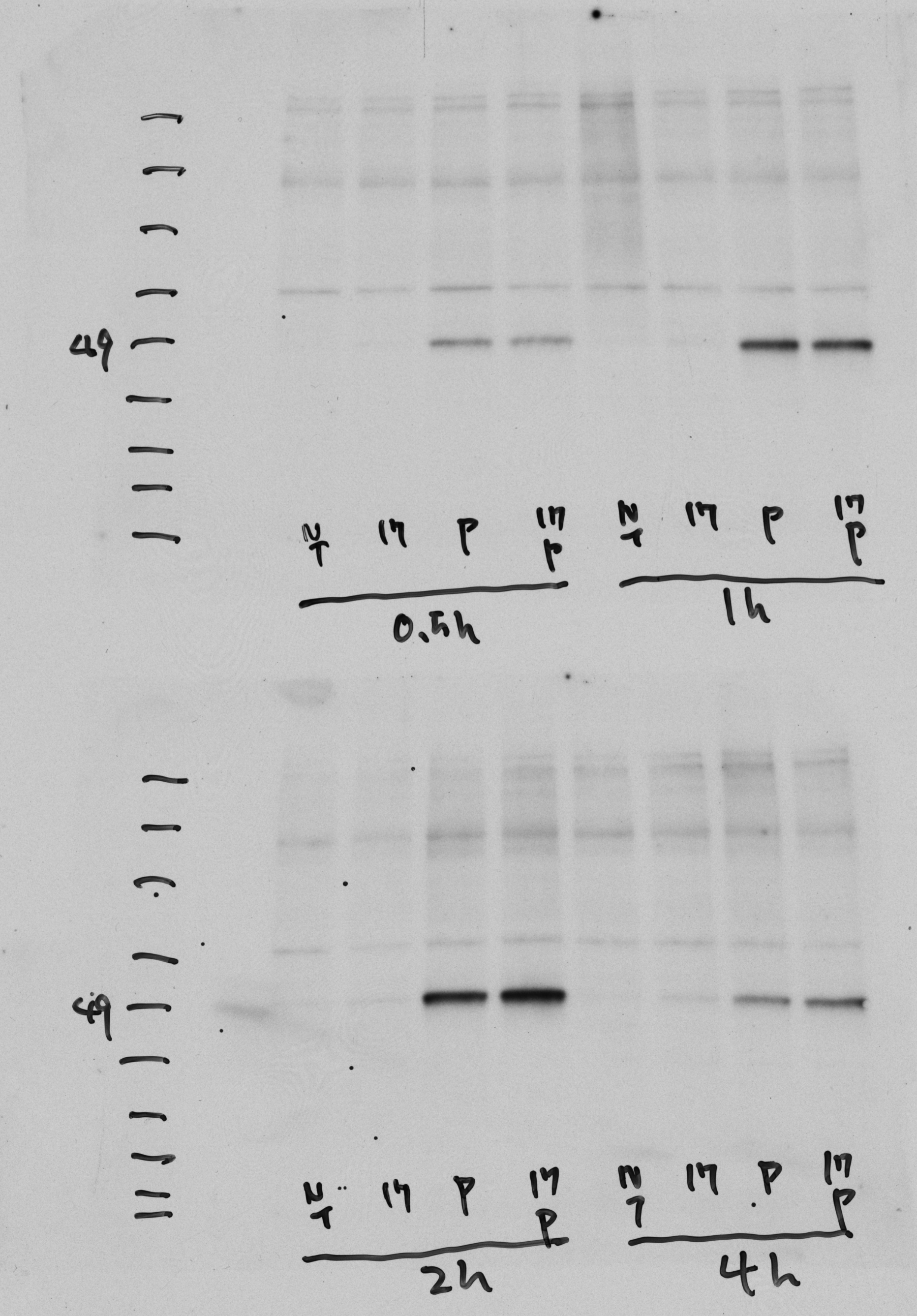

Supplement: S1 File — (TIF) [file pone.0139491.s001.tif]

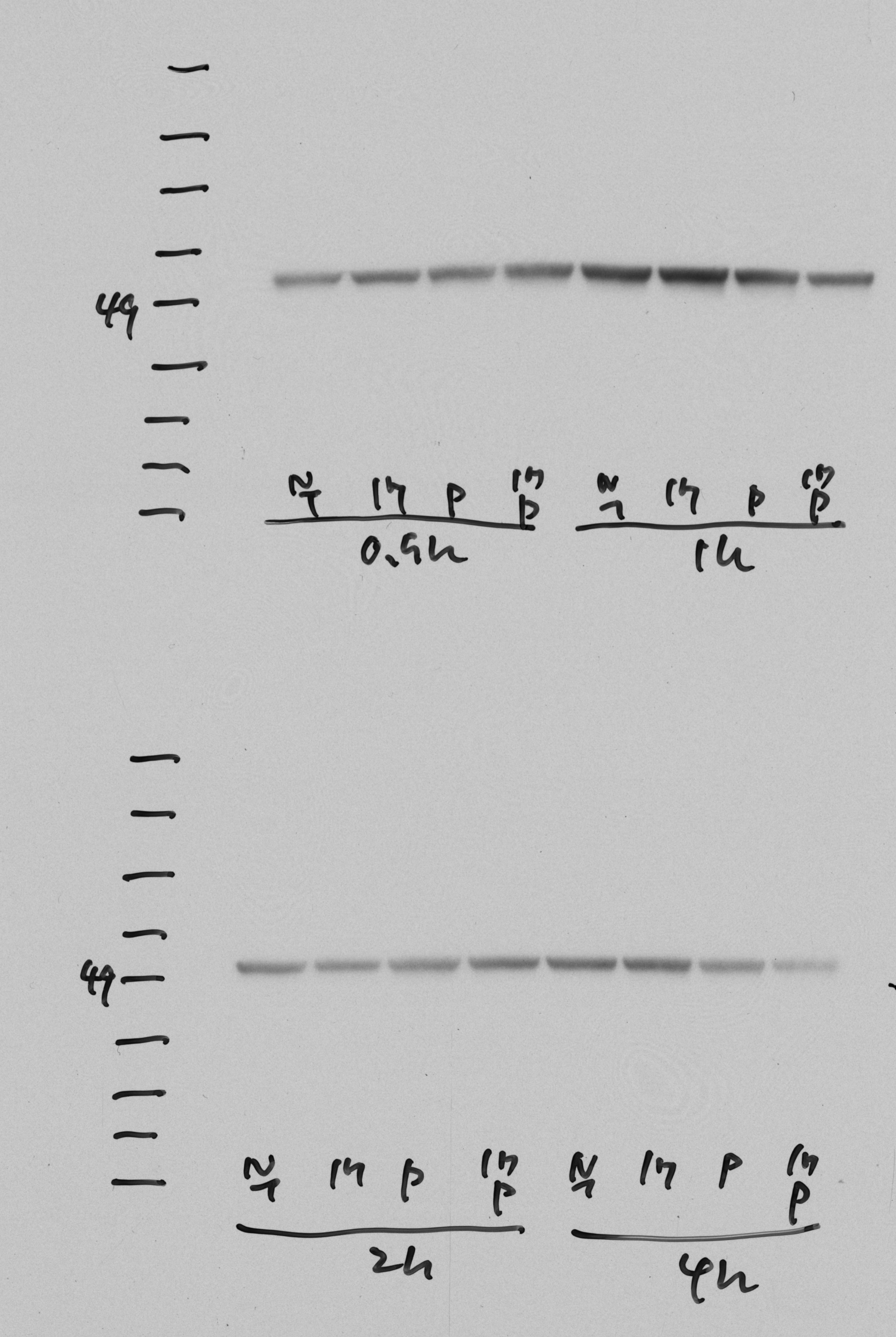

Supplement: S2 File — (TIF) [file pone.0139491.s002.tif]

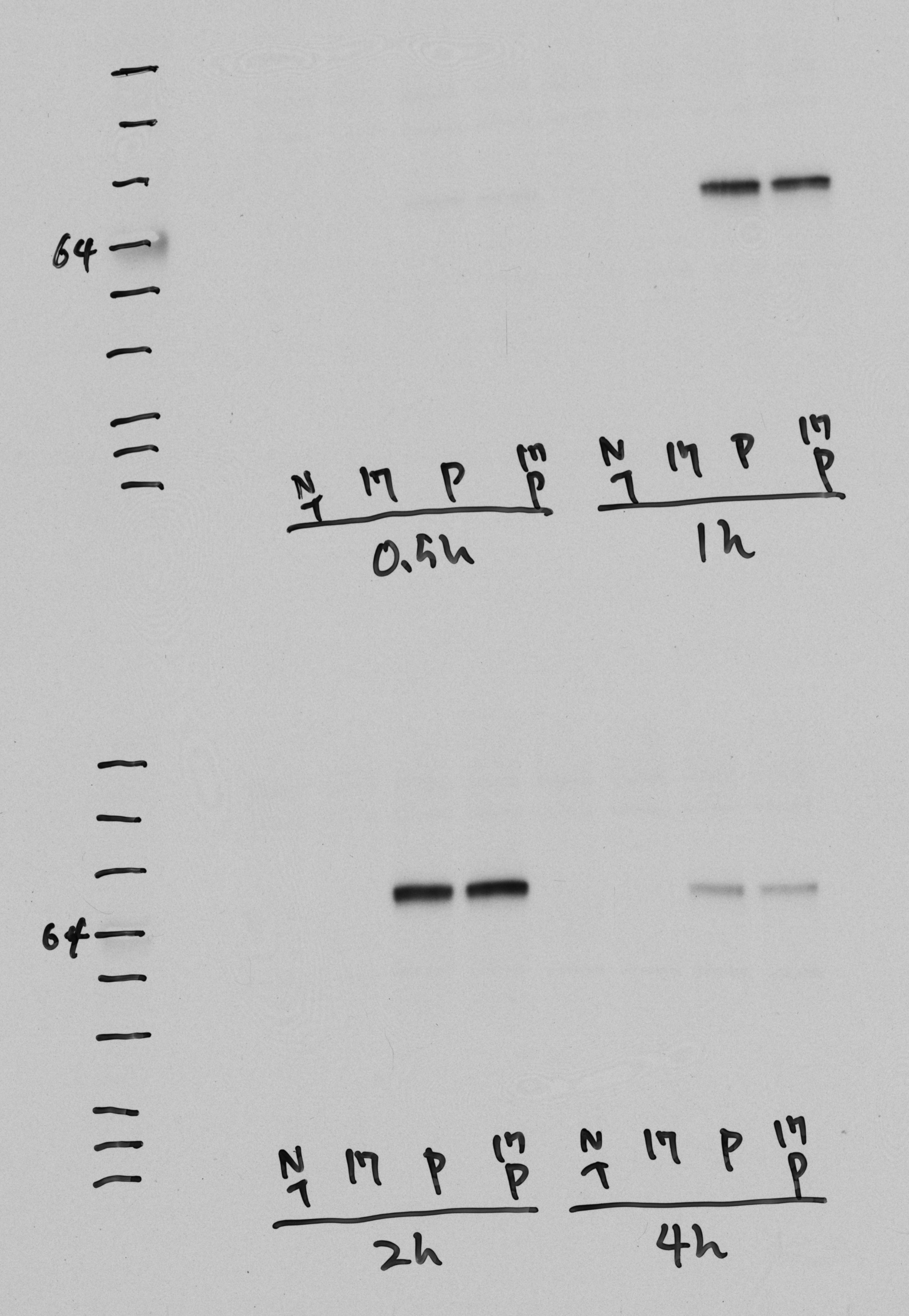

Supplement: S3 File — (TIF) [file pone.0139491.s003.tif]

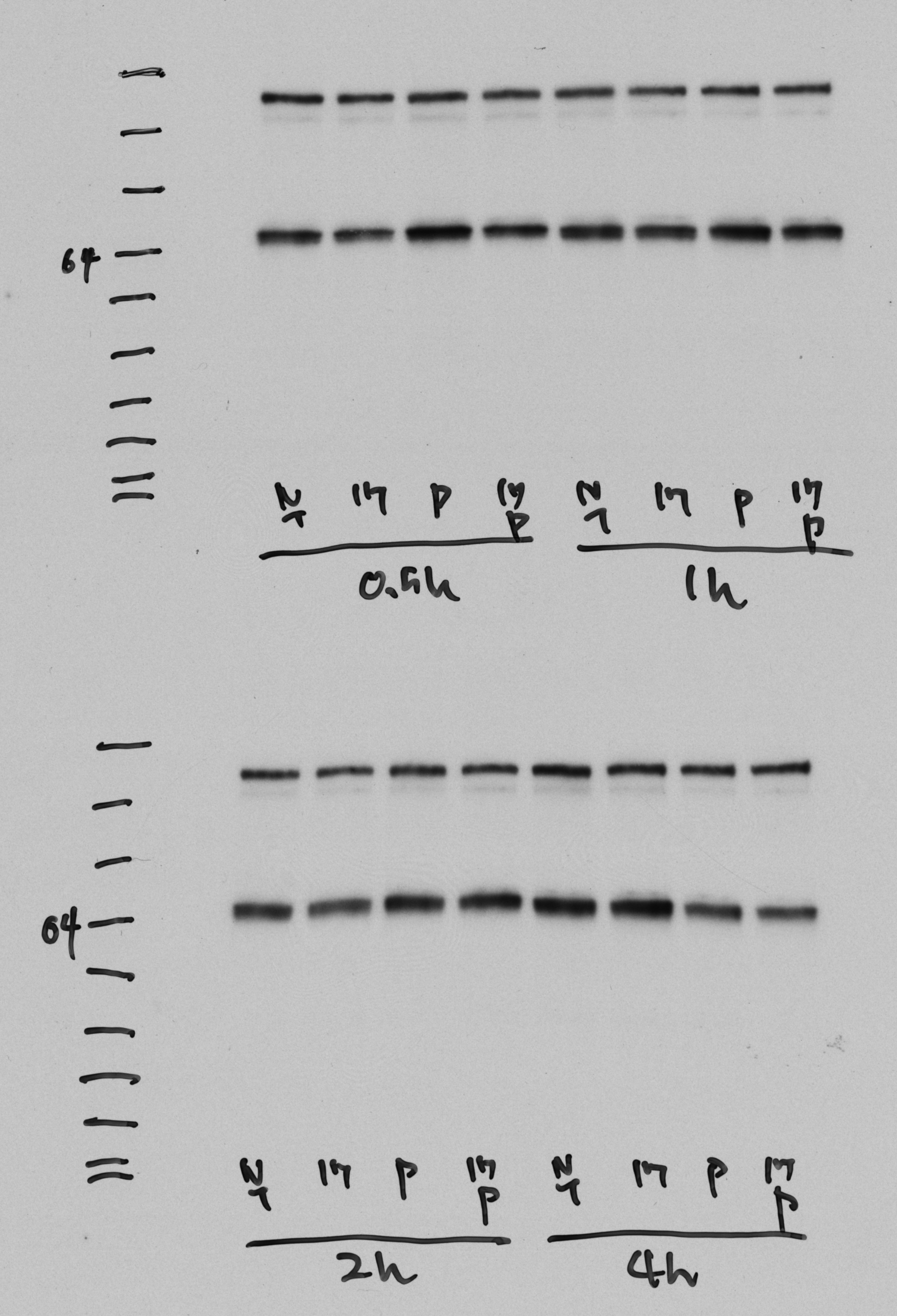

Supplement: S4 File — (TIF) [file pone.0139491.s004.tif]
