# Supplementary material for: Synergistic Proinflammatory Responses by IL-17A and Toll-Like Receptor 3 in Human Airway Epithelial Cells
Source: PLoS One. 2015 Sep 29;10(9):e0139491. doi: 10.1371/journal.pone.0139491 (PMC4587973; doi:10.1371/journal.pone.0139491)
Supplement: S1 Table — (DOCX) [file pone.0139491.s005.docx]

**S1 Table. The list of primers used in real-time RT-PCR analysis**

| **Target gene** |  | **Sequence** |
| --- | --- | --- |
| GAPDH | forward | TGGGCTACACTGAGCACCAG |
|  | reverse | GGGTGTCGCTGTTGAAGTCA |
| β-actin | forward | AGTCGGTTGGAGCGAGCAT |
|  | reverse | AAAGTCCTCGGCCACATTGT |
| G-CSF | forward | GATGGAAGAACTGGGAATGG |
|  | reverse | GACACCTCCAGGAAGCTCTG |
| IL-8 | forward | GAGAAGTTTTTGAAGAGGGCTGAG |
|  | reverse | ATCTGGCAACCCTACAACAGAC |
| CXCL1 | forward | GAAAGCTTGCCTCAATCCTG |
|  | reverse | CACCAGTGAGCTTCCTCCTC |
| CXCL5 | forward | TTTGGACGGTGGAAACAAGG |
|  | reverse | TCTCTGCTGAAGACTGGGAAAC |
| IL-1F9 | forward | GTTGGAGAACAGCCCACATT |
|  | reverse | GTTGGAGAACAGCCCACATT |
| IFN-α1 | forward | AGACTCTCACCCCTGCTATAAC |
|  | reverse | CCACAGTGTAAAGGTGCACATG |
| IFN-β | forward | TGGGAGGATTCTGCATTACCTG |
|  | reverse | TCGGAGGTAACCTGTAAGTCTG |
| TLR3 | forward | GAGGCGGGTGTTTTTGAACTAG |
|  | reverse | GCATGATGTACCTTGAATCTTTTGC |
| TICAM-1/TRIF | forward | CCCGGATCCCTGATCTGC |
|  | reverse | AGGTGGTGAAGGCATGTTCC |
| p65 | forward | AGCTCAAGATCTGCCGAGTG |
|  | reverse | ACATCAGCTTGCGAAAAGGA |
| IRF3 | forward | TTTTCCCAGCCAGACACCTC |
|  | reverse | CCAGAATGTCTTCCTGGGTATCAG |
| TNFR1 | forward | TGTTGCCCCTGGTCATTTTC |
|  | reverse | TCAAGCTCCCCCTCTTTTTCAG |
