# Supplementary material for: Synergistic Proinflammatory Responses by IL-17A and Toll-Like Receptor 3 in Human Airway Epithelial Cells
Source: PLoS One. 2015 Sep 29;10(9):e0139491. doi: 10.1371/journal.pone.0139491 (PMC4587973; doi:10.1371/journal.pone.0139491)
Supplement: S2 Table — (DOCX) [file pone.0139491.s006.docx]

**S2 Table. Ratio of the mRNA expression levels between co-treatment with IL-17A/polyI:C and polyI:C alone.**

|  |  | Amplification ratio (co-treatment / polyI:C) | |  |
| --- | --- | --- | --- | --- |
| Target of siRNA | Gene | Negative control | siRNA | P value ^*^ |
| p65 | G-CSF | 5.87 ± 0.51 | 2.29 ± 0.16 | < 0.0001 |
|  | IL-8 | 5.37 ± 0.30 | 2.62 ± 0.36 | < 0.0001 |
| IRF3 | G-CSF | 6.78 ± 0.59 | 11.0 ± 3.47 | 0.181 |
|  | IL-8 | 9.94 ± 0.91 | 5.26 ± 0.47 | 0.011 |

Amplification ratio was expressed as mean ± SE.

* Differences between co-treatment with IL-17A/polyI:C and polyI:C alone were compared using Welch’s unpaired t-test.
